# Differential effects of isoflurane on auditory and visually evoked potentials in the cat

**DOI:** 10.3389/fnsys.2024.1367525

**Published:** 2024-12-04

**Authors:** Xiaohan Bao, Paisley Barnes, Stephen G. Lomber

**Affiliations:** ^1^Integrated Program in Neuroscience, McGill University, Montreal, QC, Canada; ^2^Department of Physiology, McGill University, Montreal, QC, Canada

**Keywords:** auditory evoked potentials, visually evoked potentials, dexmedetomidine, isoflurane, cat sensory system

## Abstract

Evoked potentials can be used as an intraoperative monitoring measure in neurological surgery. Auditory evoked potentials (AEPs), or specifically brainstem auditory evoked responses (BAERs), are known for being minimally affected by anesthetics, while visually evoked potentials (VEPs) are presumed to be unreliable and easily affected by anesthetics. While many anesthesia trials or intraoperative recordings have provided evidence in support of these hypotheses, the comparisons were always made between AEPs and VEPs recorded sequentially, rather than recorded at the same time. Although the logistics of improving data comparability of AEPs and VEPs may be a challenge in clinical settings, it is much more approachable in animal models to measure AEPs and VEPs as simultaneously as possible. Five cats under dexmedetomidine sedation received five, 10-min blocks of isoflurane with varying concentrations while click-evoked AEPs and flash-evoked VEPs were recorded from subdermal electrodes. We found that, in terms of their waveforms, (1) short-latency AEPs (BAERs) were the least affected while middle-latency AEPs were dramatically altered by isoflurane, and (2) short-latency VEPs was less persistent than that of short-latency AEPs, while both middle- and long-latency VEPs were largely suppressed by isoflurane and, in some cases, completely diminished. In addition, the signal strength in all but the middle-latency AEPs was significantly suppressed by isoflurane. We identified multiple AEP or VEP peak components demonstrating suppressed amplitudes and/or changed latencies by isoflurane. Overall, we confirmed that both cat AEPs and VEPs are affected during isoflurane anesthesia, as in humans.

## 1 Introduction

For the intraoperative monitoring of anesthesia, brainstem auditory evoked potentials (BAEPs) and somatosensory evoked potentials (SSEPs) are commonly used and are preferred over visual evoked potentials (VEPs) (Sloan and Heyer, 2002; Banoub et al., 2003). Over three decades ago, it was demonstrated that the amplitude of VEPs and cortical SSEPs (Sebel et al., 1984; Sebel et al., 1986) but not BAEPs (Sebel et al., 1986) are decreased during surgical level anesthesia. The same effect of anesthetics on VEP amplitudes was also shown by more recent studies (Chi and Field, 1990; Wiedemayer et al., 2003; Tenenbein et al., 2006; Tanaka et al., 2020). The susceptibility of VEPs to anesthetics has brought challenges to some surgical procedures where the monitoring of intact visual function is essential, such as surgery to remove a cancerous tumor near the optic nerve. Moreover, the confounding effect of anesthetics has long been a persistent concern for neuroscientists studying sensory functions in anesthetized animals, and has motivated many investigations on this issue (Cheung et al., 2001; Santangelo et al., 2018).

The vast majority of retinal ganglion neurons are subcortically relayed in the lateral geniculate nucleus (LGN) with a minority (less than 10%) projecting to superior colliculus (Perry and Cowey, 1984). It is now generally recognized that sub-cortical nuclei along ascending sensory pathways are less affected by anesthetics, or that neurons distant from the cortex are less affected by anesthetics. While halothane and enflurane suppressed cortically generated SSEPs measured from a central C3 electrode with reference electrode at forehead, they did no suppress cervically generated SSEPs from a spinal C2 electrode (Samra et al., 1987; Sebel et al., 1987). Similarly in the auditory system, the amplitude of auditory middle latency responses (MLRs), which is identified to be generated by the auditory thalamus and primary cortical areas, was decreased by propofol or isoflurane (Schwender et al., 1994a); this was not the case for BAEPs (Manninen et al., 1985; Sebel et al., 1987).

One of the technical features that are particular to AEP recordings is that AEPs originating from cortical, thalamic, midbrain, and brainstem sources can be recorded using the same electrode configuration (Caron-Desrochers et al., 2018). This electrode configuration allows VEP recordings as well. Taking this feature as an advantage, it is possible to compare simultaneously-recorded BAEPs, MLRs, and cortical AEPs (cAEPs). The use of simultaneous recording minimizes the confounding factors or variations introduced by different subjects, different electrodes, and different levels of anesthesia.

**Figure 1:**
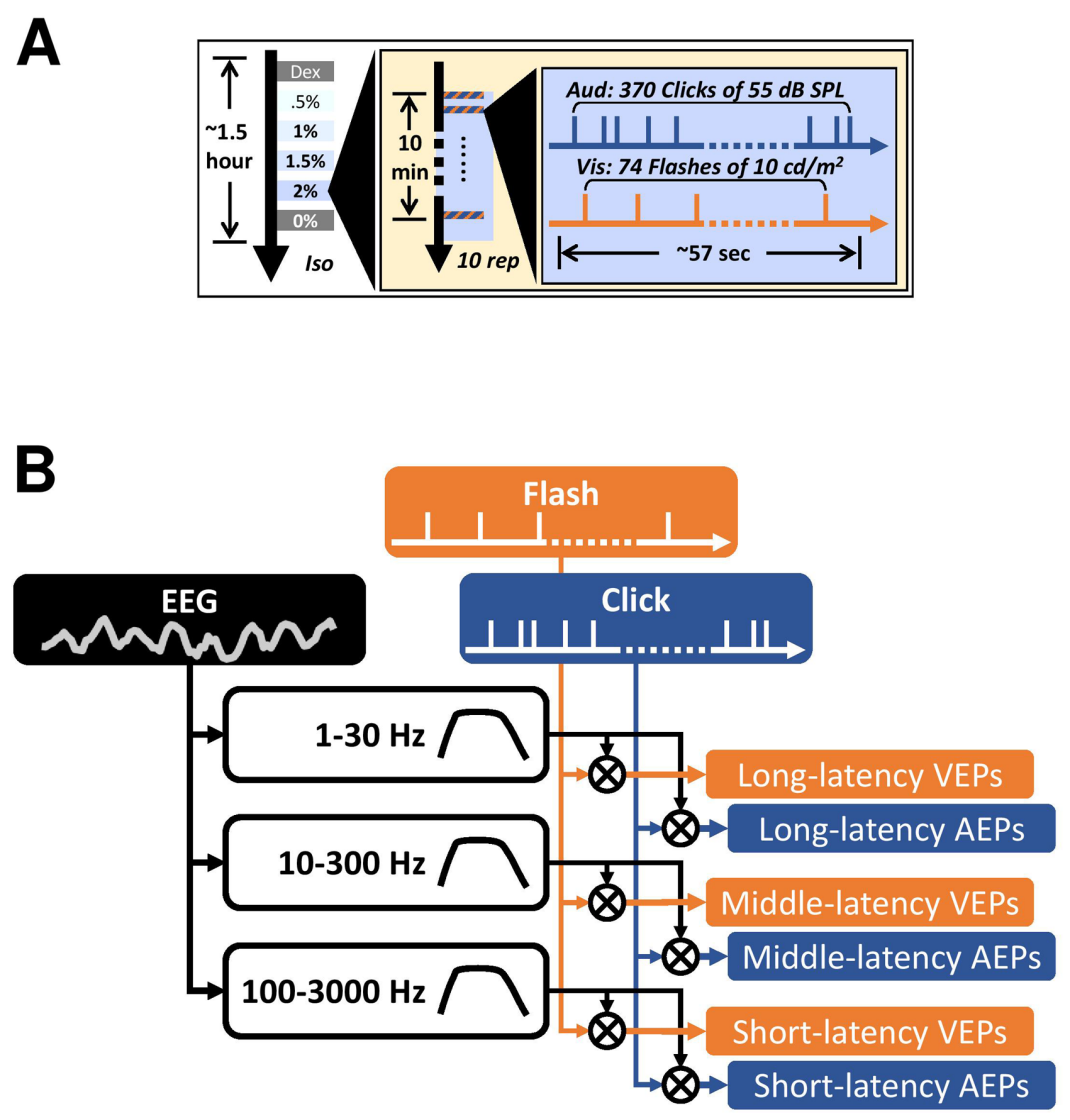
Experiment design and data analysis. **(A)** The timeline of the auditory and the visual stimulus trains used in the current study. The three concentric boxes contain the timeline of each trial, each block, and the entire recording in each subject, respectively. Dex, dexmedetomidine-only block. Iso, isoflurane. Rep, repetition. **(B)** An illustration of the data analysis pipeline on deriving the AEPs and the VEPs. The cross inside a circle indicates cross-correlation. All filled boxes are signals. The unfilled boxes indicate digital filters with varying pass-bands.

In this study, we anesthetized five cats under four different isoflurane concentrations and performed 10 EEG recording trials for each concentration. Stimulus presentation for one recording trial comprised a 57-s train of asynchronized click and flash pulses (Figure 1). EEG signals were filtered off-line using three different band-pass filters to acquire VEPs and AEPs in a wide range latency window, which we referred to as short-latency (SL-), middle-latency (ML-), and long-latency (LL-) AEPs/VEPs. The filter and epoch parameters were mainly derived from the convention for auditory brainstem responses (ABRs), middle-latency responses (MLRs), and late auditory evoked potentials (Picton, 2010). AEPs composed of these subcomponents, in general, reflect neural activities in response to sounds along the ascending auditory pathway.

In addition to quantifying the overall signal strength of the waveform as the major focus of this study, peak components were also measured. In human evoked potentials, some of those components were better-known for their sources of generator, e.g., cochlear nucleus for P3 in SL-AEPs, inferior colliculus for P5 in SL-AEPs (Hashimoto et al., 1981; Picton, 2010), their developmental properties, e.g., reduced latency of N1 in LL-AEPs (Sharma et al., 1997), and the interpretations of polarity, e.g., opposite polarity LL-VEPs for stimuli in upper versus lower visual fields (Creel, 2019), many others were not. Knowing these properties under isoflurane anesthesia would also contribute to a more complete profile for those components. This stimulus paradigm allowed us to compare the dynamics of isoflurane effect along cat auditory and visual pathway as reflected in EEG signal with minimized confounding variables. We hypothesized that visually evoked potential receive a greater suppression than auditory evoked potential during isoflurane anesthesia, and it is true even if the both were recorded simultaneously and processed with the same filter. We found that both AEPs and VEPs were affected by isoflurane anesthesia. While VEP magnitude was overall suppressed, AEP magnitude was preserved but with the waveforms largely altered.

## 2 Materials and methods

All procedures were conducted in compliance with the National Research Council’s Guide for the Care and Use of Laboratory Animals (8th edition; 2011) and the Canadian Council on Animal Care’s Guide to the Care and Use of Experimental Animals (Canadian Council on Animal Care 1993). Furthermore, the following procedures were approved by Animal Care Committee (DOWB) for the Faculty of Medicine and Health Sciences at McGill University.

### 2.1 Animal preparation and anesthesia protocol

Five cats (3-to-5 years old, 4 females) were used in this study. After subject was sedated by 0.04 mg/kg dexmedetomidine (Dexdomitor, Zoetis) injected intramuscularly, the left eye was occluded using a black contact lens so that visual stimuli were presented unilaterally. Phenylephrine (Mydfrin, Alcon) was applied to the right eye to dilate the pupil, and saline drops were used as lubrication. Subjects were placed on a water-circulated heating pad (TP-400, Gaymar) with their nose and mouth covered by an anesthesia mask. Subjects were not intubated, because most of recording was conducted under light anesthesia, whereby intubation may cause discomfort. Given that the subjects were recovered in the end, we did not use a tracheotomy. Throughout the recording, breathing was unassisted. Once heart rate and SpO2 were stable, two 15-min recording blocks were carried out while the subject was breathing 80% oxygen (Dispomed) as a baseline under dexmedetomidine, i.e., Dex condition. Next, the oxygen was mixed with isoflurane (AErrane, Baxter) at four inspired concentrations (0.5, 1, 1.5, and 2%, as a “wash-in” period) using a vaporizer (Isotec 4, Smiths Medical). Given the wash-in process of isoflurane as an inhalational agent (Torri et al., 2002), a 10-min recording block was carried out for each concentration (except for subject No. 1 each recording block was 15 min). Finally, another 10-min block was recorded after the termination of isoflurane, i.e., “0%” condition as a “wash-out” period, to investigate the effect of anesthesia recovery (Figure 1A). Subject’s vital signs and electrode impedance were checked at the end of each recording block. Each recording block started immediately after changing the concentration setting on the vaporizer. At the end of data collection, electrodes and contact lens were removed before the atipamezole (Antisedan, Zoetis) was administrated intramuscularly to facilitate recovery from dexmedetomidine sedation.

### 2.2 Visual and auditory stimuli

The visual stimuli consisted of flashes presented to subjects from a 5-mm-diameter light-emitting diode (~11 degrees of visual field, LED, DigiKey). The flash was calibrated to 10 lux in intensity in dark and presented under photopic condition during recording. The auditory stimuli were clicks emitted by an 8-cm-diamter loudspeaker (Fostex), calibrated to 55 dB SPL (Model 2250, B&K). Both auditory and visual stimulation signals were generated by the same processor (RZ2, TDT). The LED was attached to the top of the loudspeaker and placed 8-cm away from the subject 45 degrees to the right of the midline.

To manipulate the timing of auditory and visual stimuli, two independent, 57-s-long pulse trains for triggering clicks and flashes, respectively, were pre-made in Matlab using a Poisson random process and loaded into the stimulus/recording software (Synapse, TDT). The auditory stimulus train contained 370 click pulses and the visual stimulus train contained 74 flash pulses. A second click was not allowed during the period of 20 ms after the preceding click, as defined as an inter-click deadtime. Similarly, inter-flash deadtime was set to 300 ms. On average, the inter-stimulus intervals (ISIs) were 159 ms for clicks and 717 ms for flashes. The Poisson random process was previously used to characterize the properties of neurons in cat primary auditory cortex (Barbour and Wang, 2003; Gourévitch and Eggermont, 2008). The use of Poisson random process instead of a fixed interstimulus interval ensure that there is no correspondence between the auditory and the visual stimuli (Rabinowitz et al., 2011; Valentine and Eggermont, 2004). The exact same stimulus presentation was repeated 10 trials under each isoflurane conditions (Dex, 0.5, 1, 1.5, 2, 0%, in order) at a rate of ~1 trial per minute (Figure 1A).

### 2.3 EEG recording and signal processing

Stainless-steel needles (25G) were placed subdermal as recording electrodes. The active electrode was placed near the midpoint of subject’s interaural line, while the reference electrode was placed beneath the right ear. The ground electrode was placed on subject’s dorsum. The impedance of both active and reference electrodes was maintained below 3 kOhm during recording. The signal was amplified and digitized with a pre-amplifier (TDT, Medusa4Z) at ~6.1 kHz, streamed onto a digital signal processor (TDT, RZ2), and stored on a computer hard drive.

Signal was digitally notched offline to remove 60-Hz noise before passing through three different band-pass filters (1–30 Hz, 10–300 Hz, and 100–3,000 Hz) for long-, middle-, and short-latency responses, respectively (Figure 1B). For short-latency responses, the original signal was upsampled to ~24.4 kHz before filtering. Epochs of various windows were extracted surrounding either click or flash onsets, and averaged to derive auditory or visually evoked potentials.

### 2.4 Data analysis

Root-mean-square values were obtained using MATLAB built-in function *rms()*. Each averaged waveform was separated into a pre-stimulus zero-mean baseline window and a post-stimulus response window, producing two RMS values, respectively. The corrected RMS value was calculated as in the following equation:


$${RMS}_{\mathrm{corrected}}=\sqrt{{{RMS}_{\mathrm{response}}}^{2}-{{RMS}_{\mathrm{baseline}}}^{2}}$$

A customized algorithm was used to determine and to analyze individual components of response waveforms.

### 2.5 Statistics

Due to a small sample size (*N* = 5) in this study, the Friedman test was chosen to examine the statistical significance of difference among isoflurane blocks. Post-hoc pairwise comparisons were carried out when there was a significant main effect (*p* < 0.05) using Tukey’s honestly significant difference procedure. To manage the potential increase in Type I error, false discovery rates (FDRs) were estimated in a permutation test. The same multiple comparison test used on the original data was performed 1,000 times where the data were randomly designated to one of the six isoflurane blocks independently for each subject. The *p*-values derived from 15 pairwise comparisons across all permutations were pooled together to estimate the probability of detecting a significant difference (i.e., false positive), which is also known as q-value. In each subject, we also examined the effect of isoflurane blocks measurements derived from trial averages using Kruskal–Wallis test. Both tests are non-parametric versions of one-way analysis of variance (ANOVA), and are available in Statistics and Machine Learning Toolbox™ on MATLAB as *friedman()*, *kruskalwallis()* and *multcompare()*. Comparisons of curve fitting coefficients were examined by Wilcoxon signed rank test using MATLAB function *signrank()*.

## 3 Results

The effect of anesthetics on electroencephalogram (EEG) signals without sensory inputs have been widely reported (for review, see Bruhn et al., 2006). Among those classic EEG measurements previously used, we picked the Power Spectral Density (PSD) and the Approximate Entropy (ApEn) to further look into. We performed these analyses before reporting the sensory-related components in EEG as our major focus of the current study.

Previous studies including Power Spectral Density (PSD) analysis focused on frequency below 50 Hz (Akeju et al., 2014; Kreuzer et al., 2020; Findeiss et al., 1969), while our analysis included frequencies up to 3 kHz. As shown by Welch’s PSD estimate, the energy of the EEG signal was heavily distributed at low frequency and diminished as the frequency increased. This pattern was highly consistent under different isoflurane conditions (Supplementary Figure S1). Compared to the dexmedetomidine baseline, a decrease in EEG power was observed between 30 Hz and 100 Hz in the 1.5%- and the 2%-isoflurane blocks, while an increase in EEG power was observed in the frequency range between 100 Hz and 300 Hz.

Approximate Entropy (ApEn) is a representative of a variety of time-domain entropy indices (Liang et al., 2015; Bruhn et al., 2003). One of the pioneering studies has established an association between ApEn and desflurane concentrations (Bruhn et al., 2000). We calculated the ApEn values for different isoflurane conditions in each of the three bandpass filtered EEG signals. The most prominent ApEn change was found in the 10–300 Hz band (Supplementary Figure S2), where the median of ApEn values decreased with the up-stepping isoflurane concentration systematically and returned toward baseline after the termination of isoflurane.

### 3.1 Effect of isoflurane on waveforms and signal strength

**Figure 2:**
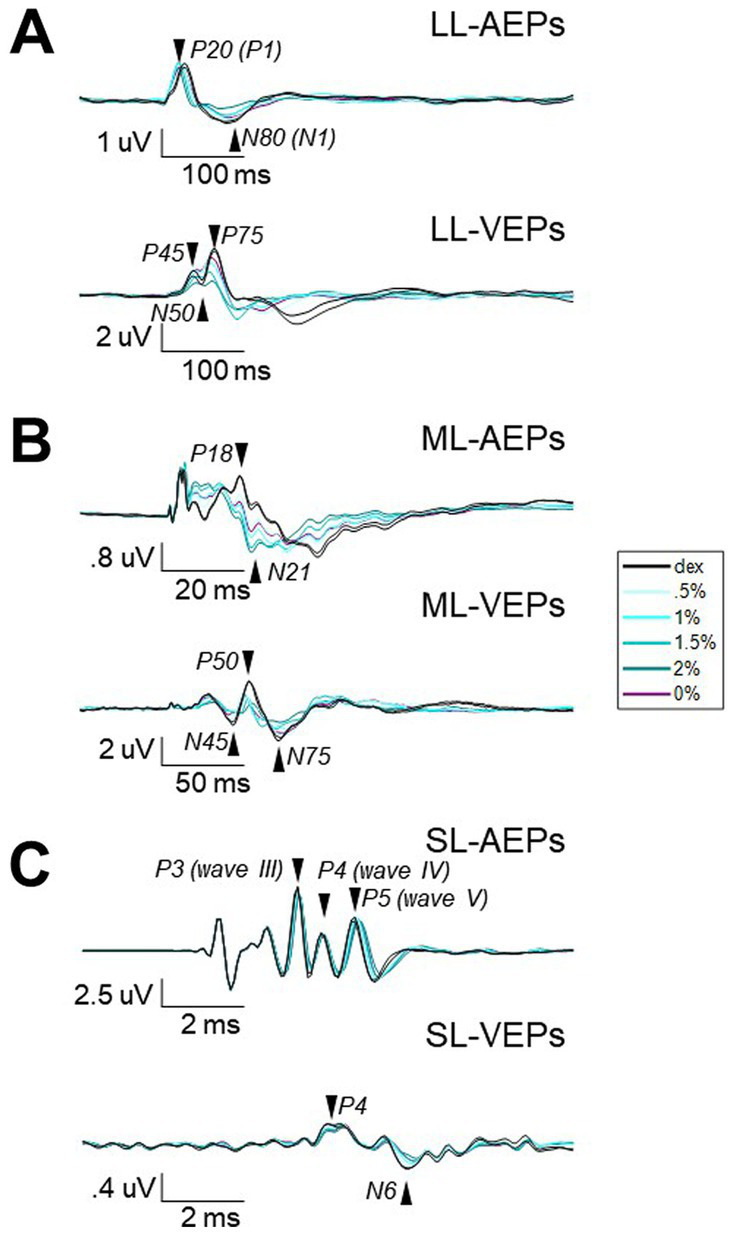
The averaged waveforms of AEPs and VEPs from three different filters. Waveforms of AEPs or VEPs averaged across stimulus presentations and subjects for long-latency **(A)**, middle-latency **(B)**, and short-latency **(C)**. Note that different time and amplitude scales were used. Vertical scaler is aligned to the stimulus onset. The isoflurane/anesthesia treatment is color-coded. Black, baseline block with only dexmedetomidine (i.e., Dex) administrated. Blue with light-to-dark shading, the isoflurane blocks with up-stepping concentrations. Purple, the block after isoflurane stops. Arrows and labels indicate the selected peak components for further analysis.

**Figure 3:**
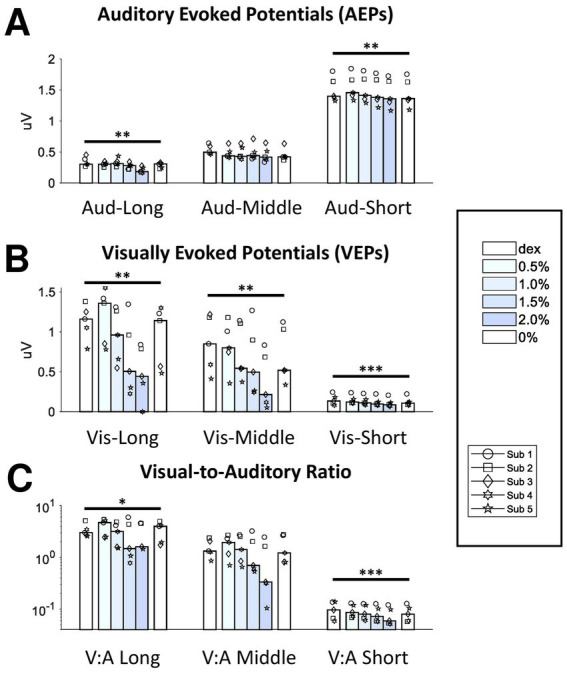
Effect of isoflurane on the signal strength of AEPs and VEPs. The bottom axis shows the block median of the RMS values across subjects from 6 blocks filled with different shading of blue indicating isoflurane concentration. Dex, dexmedetomidine-only block. From the left to right, the data from the three filters were clustered for AEPs **(A)** and VEPs **(B)**. The RMS ratios between VEPs and AEPs were also shown here **(C)**. Dots with different shapes, data of individual subject. *p < 0.05, **p < 0.01, ***p < 0.001.

To evaluate the effect of isoflurane, we will first compare signal strength quantified from the entire waveform among blocks (Figures 2, 3; Table 1; Supplementary Figures S3–S8). While the narrative in this study mainly focuses on the population level using the block-averaged waveforms from individual subjects, the trial-averaged waveforms were also analyzed to reveal the trend of isoflurane effects in each subject. A selected set of individual peak components were also examined. The effect of isoflurane peak amplitude and peak latencies in these components are summarized (Table 2) in terms of their change during the period of ascending isoflurane concentration (i.e., during the “wash-in” period) and the recovery block (i.e., during the “wash-out” period). Details on individual peak measurements were not included as the main results of our findings (Supplementary Figures S9–S14), although there are interesting findings with some of these measurements. For example, the isoflurane effect observed with P18 and N21 peak components that are derived from ML-AEPs was different the isoflurane effect observed in the RMSs of ML-AEPs. For example, despite of no change of signal strength for MLAEPs during isoflurane administration indicated the corrected RMS measure, the reformation of the waveforms was clearly captured using a couple of peak measurements.

**Table 1:** Multiple comparisons between isoflurane blocks for RMS values.

Cut-off frequency	Stimulus modality	Comparison pair	Significance level	False discovery rate
1–30 Hz	Auditory	2%-isoflurane versus dex block	*p =* 0.005 < 0.01	*q =* 0.0001 < 0.001
		2%- versus 1%-isoflurane block	*p =* 0.009 < 0.01	*q =* 0.0005 < 0.001
	Visual	2%-isoflurane versus dex block	*p =* 0.028 < 0.05	*q =* 0.0007 < 0.001
		2%- versus 0.5%-isoflurane block	*p =* 0.005 < 0.01	*q* < 0.0001
10–300 Hz	Auditory	No main effect	n/a	n/a
	Visual	2%-isoflurane versus dex block	*p =* 0.009 < 0.01	*q =* 0.0002 < 0.001
		2%- versus 0.5%-isoflurane block	*p =* 0.028 < 0.05	*q =* 0.0018 < 0.01
		2%- versus 1%-isoflurane block	*p =* 0.047 < 0.05	*q =* 0.0041 < 0.01
100–3,000 Hz	Auditory	2%- versus 0.5%-isoflurane block	*p =* 0.005 < 0.01	*q* < 0.0001
		2%- versus 1%-isoflurane block	*p =* 0.047 < 0.05	*q =* 0.0030 < 0.01
	Visual	2%-isoflurane versus dex block	*p =* 0.005 < 0.01	*q* < 0.0001
		2%- versus 0.5%-isoflurane block	*p =* 0.003 < 0.01	*q* < 0.0001
		2%- versus 1%-isoflurane block	*p =* 0.047 < 0.05	*q =* 0.002 < 0.01

**Table 2:** A list of peak components and isoflurane effects.

Cut-off frequency	Stimulus modality	Peak component	Amplitude Wash-in/Wash-out		Peak time Wash-in/Wash-out	
1–30 Hz	Auditory	P20	–	–	↓	–
		N80	↓	–	–	↑
	Visual	P45	–	↑	–	–
		N50	–	↑	–	–
		P75	–	↑	–	–
10–300 Hz	Auditory	P18	↓	–	↓	–
		N21	↑	–	–	–
	Visual	N45	–	–	–	–
		P50	↓	–	↑	↓
		N75	↓	↑	–	–
100–3,000 Hz	Auditory	P3	–	–	↑	↑
		P4	–	↓	↑	–
		P5	↓	–	↑	↑
	Visual	P4	–	–	↑	↑
		N6	↓	–	↑	↑

Each long-latency AEP waveform was characterized by a main positive peak component with a peak time around 20-ms (P20) followed by a less prominent but observable negative component around 80-ms (N80). The administration of isoflurane did not abolish LL-AEPs (Figure 2A, Top; Supplementary Figure S3), but consistently decreased the peak time of P20. The effect of isoflurane on Root Mean Square (RMS) values, which serves as a quantification of signal strength, was small but significant (*Q* = 13.57, *p* = 0.003 < 0.01; Figure 3A, Left). Using the same filter and window parameters, we observed long-latency (LL-) VEPs in all five subjects as well. The effect of isoflurane on LL-VEPs was overall suppressive but dependent on isoflurane concentration (Figure 2A, Bottom; Supplementary Figure S4). The main block effect on RMS values of LL-VEPs was statistically significant (*Q* = 16.89, *p* = 0.005 < 0.01) (Figure 3B, Left). From both the waveforms and the RMS values, only the 2%-isoflurane block showed substantial attenuation of LL-VEPs across all five subjects. In subject No. 4, LL-VEP was completely absent during the 2%-isoflurane block.

With EEG signals band-pass filtered between 10 Hz and 300 Hz, we observed middle-latency (ML-) AEPs (sometimes referred as MLAERs) in all five subjects. The administration of isoflurane dramatically altered the ML-AEP waveforms (Figure 2B, Top; Supplementary Figure S5). It is worth noting that the overall signal strength was not affected despite the alteration of the waveform. There was no statistically significant effect of isoflurane block on the RMS values (Figure 3A, Middle). Using the same filter but a longer epoch window, the most identifiable peak components (N45, P50, and N75) observed in middle-latency (ML-) VEPs had similar latencies to those in LL-VEPs but with their polarities inverted. The effect of isoflurane on ML-VEPs was quite similar to LL-VEPs (Figure 2B, Bottom; Supplementary Figure S6). The RMS values of ML-VEPs were overall significantly decreased (*Q* = 15.86, *p* = 0.007 < 0.01) (Figure 3B, Middle).

The waveforms of short-latency (SL-) AEPs had highly consistent pattern, which was composed of five distinct pairs of positive and negative peak components. Their peak times were comparable with those in the literature (Fullerton et al., 1987). The effect of isoflurane on SL-AEP waveforms was small but prominent and consistent across subjects (Figure 2C, Top; Supplementary Figure S7). The later components (e.g., wave IV and V) were delayed and prolonged, while the earlier components seemed minimally affected. Although the RMS values were decreased for less than 7% on average, the effect of isoflurane block was statistically significant (*Q* = 19.51, *p* = 0.002 < 0.01) (Figure 3A, Right). Using the same filter, we identified a positive (P4) and a negative (N6) peak component around 4-msec and 6-msec after flash onsets. The pattern of the peak components was more consistent than ML- and LL-VEPs across all five subjects, except that the N6 component had a later peak time in subject No. 4. The effect of isoflurane on this so-called short-latency (SL-) VEPs was comparable to SL-AEPs (Figure 2C, Bottom; Supplementary Figure S8) but with a larger suppression. The later component N6 was delayed and prolonged by isoflurane more than the earlier component P4. Despite of being the lowest among all six evoked potentials, the RMS values of SL-AEPs was decreased significantly (*Q* = 22.26, *p* < 0.001) (Figure 3B, Right).

Although auditory and visual systems are inherently different and difficult to compare directly, we calculated the visual-to-auditory (Vis-Aud) ratios in the RMSs for statistical analysis (Figure 3C). It was shown that the administration of isoflurane significantly decreased the Vis-Aud ratios for short- (*p* < 0.001) and long- latency (*p* = 0.046 < 0.05) responses. We concluded that the suppressive effect of isoflurane on VEPs was out of proportion with AEPs using the current stimulus paradigm, despite the fact that using different stimulus configuration (e.g., inter-stimulus intervals and intensities) is likely to lead to different results completely.

### 3.2 Peak amplitude and peak time

RMS value is a quantification applied to the entire waveform and requires minimal arbitrary supervision except for the selection of epoch window. The change of RMS value does not indicate which peak component(s) are specifically affected during the experiment. Therefore, we further examined the effect of isoflurane by selecting at least two peak components from each of the six AEPs/VEPs. The effect of isoflurane on the amplitude and latency of individual peak components were illustrated and summarized (Table 2; Supplementary Figures S9–S14). All the six AEPs or VEPs showed some changes during isoflurane anesthesia that were statistically meaningful when choosing certain quantifications and components.

### 3.3 Wash-out effect in ML-AEPs

**Figure 4:**
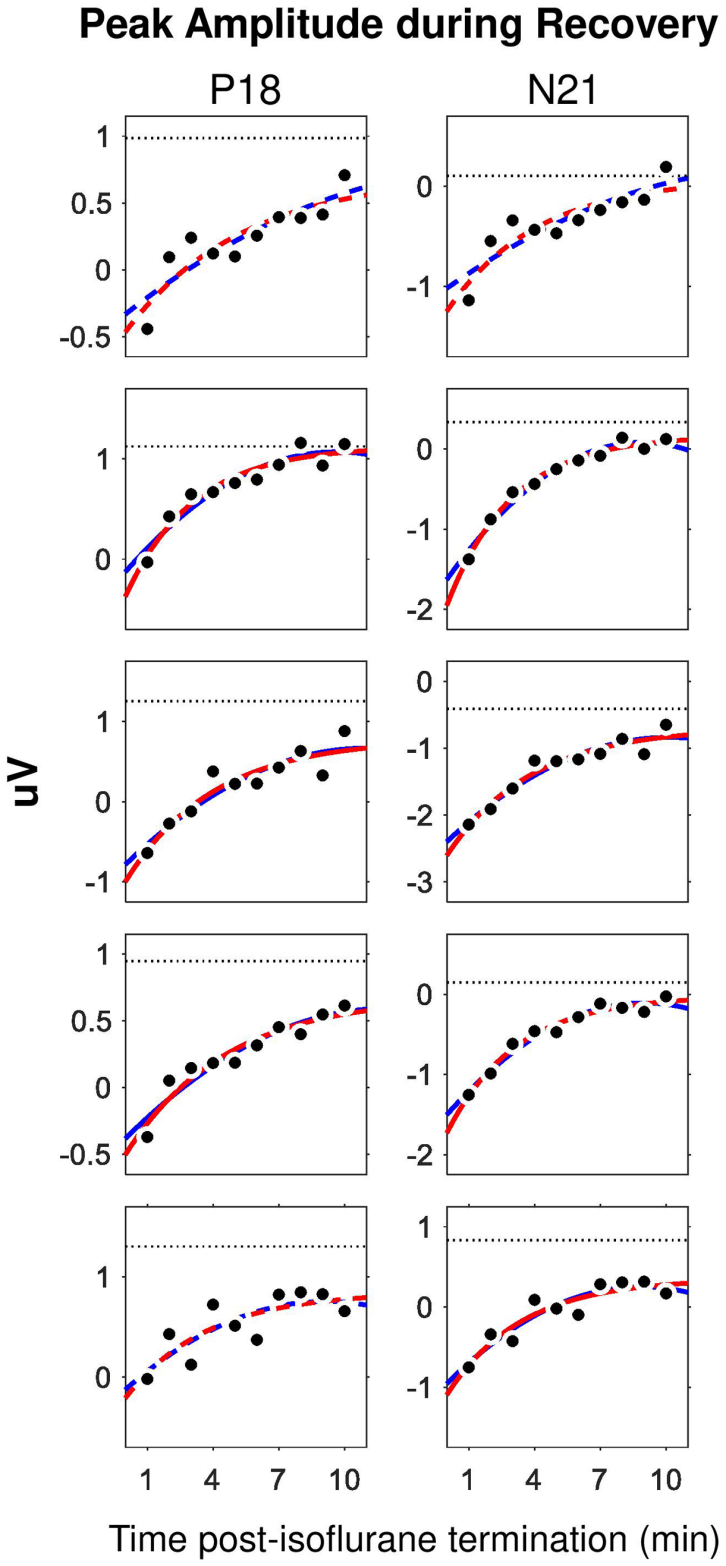
Time course of the wash-out effect in the ML-AEP components P18 and N21. Each row corresponds to one subject. Dot, peak amplitude derived from trial-averaged waveforms in the 0%-isoflurane block. Dotted line, data from the 0.5%-isoflurane block. Red, exponential mode. Blue, second order polynomial model. Solid, adjusted *R*^2^ larger than 0.75. Dashed, adjusted *R*^2^ between 0.5 and 0.75.

Taking the advantage of the small trial-by-trial variance of P18 and N21 amplitudes derived from ML-AEPs, we examined the recovery process (i.e., wash-out effect) of isoflurane during the 0%-isoflurane block by plotting these two measurements as a function of time after isoflurane termination. We found that the minute-by-minute change in both measurements may dictate the time course of the isoflurane wash-out process (Figure 4). After the termination of isoflurane administration, the recovery from anesthesia should follow an exponential decay pattern while isoflurane was cleared out of the central neural system (Stoelting and Eger, 1969). As expected, in this 10-min recovery block, both P18 and N21 peak amplitudes recovered back toward the baseline level faster during the first 5 min than the second 5 min. In some subjects, the recovery function approached a plateau in the end.

We carried out curve fitting for the recovery functions using both an exponential model, i.e., ${amp}_{\mathrm{peak}}=a\cdot e^{b\cdot \mathrm{time}}+c$ , and a second-order polynomial model, i.e., ${amp}_{\mathrm{peak}}=a\cdot {\mathrm{time}}^{2}+b\cdot \mathrm{time}+c$ . Both models have three coefficients. Both nonlinear models fit well with data. Considering that the exponential decaying pattern of the effective concentration is featured for common inhaled gas anesthetics, we only derived the coefficients from the exponential model for further comparison. In the exponential model, the coefficient a represents for a gain factor. The coefficient b represents for a decay factor. The coefficient c represents for an asymptotic value. We found no statistically significant difference for any one of the three coefficients between the P18 and the N21 components.

### 3.4 Peak III-to-V latency in SL-AEPs

**Figure 5:**
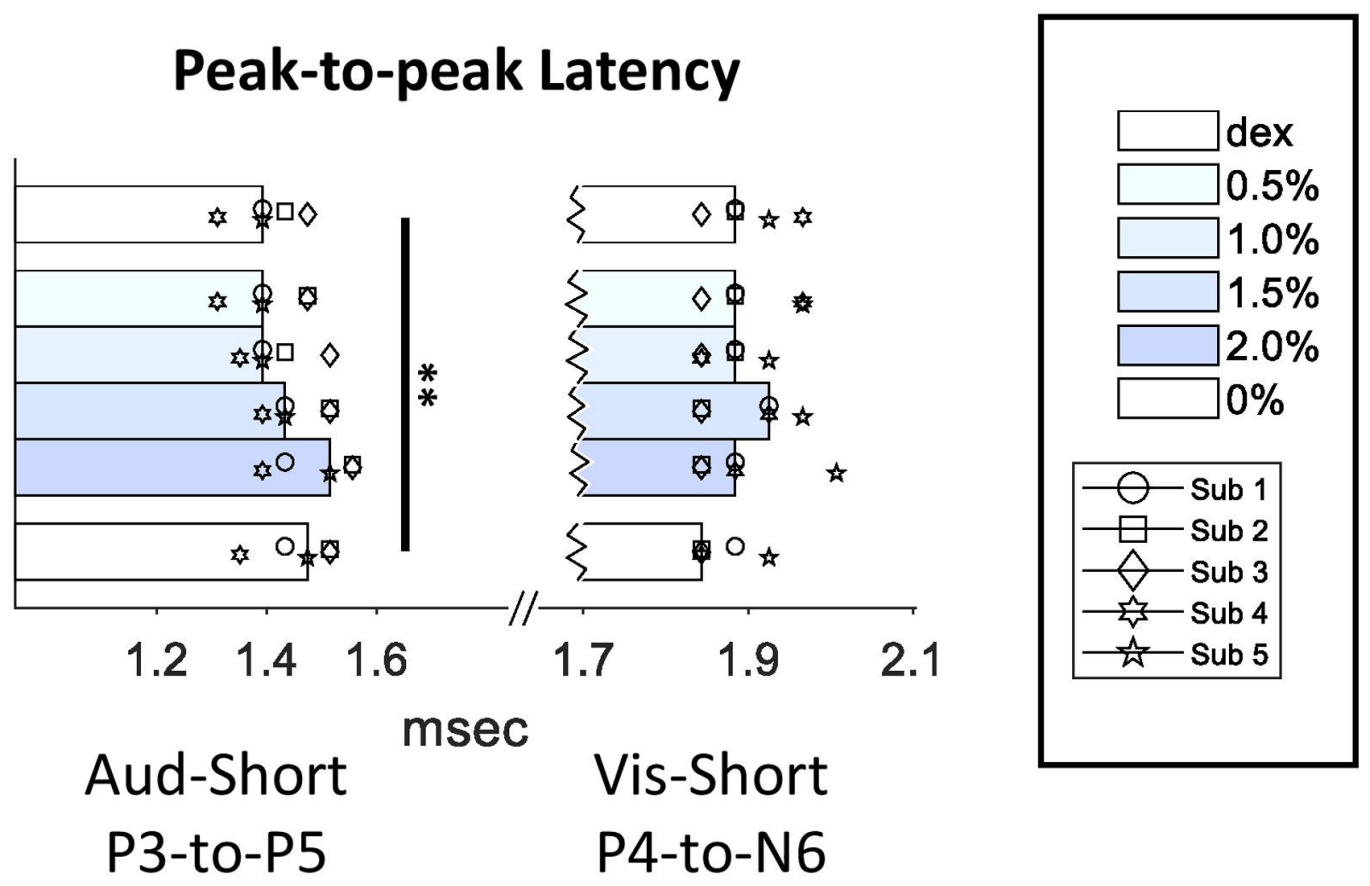
Effect of isoflurane on the peak-to-peak latencies in SL-AEPs and SL-VEPs. For conventions, see Figure 3 legend. Multiple comparison test showed a significant increase in P3-to-P5 latency in the 2%-isoflurane block when compared to the Dex (*p* < 0.01) and the 0.5%-isoflurane block (*p* < 0.05).

In previous studies, peak III-to-V latency was frequently used as a quantification for intraoperative ABR monitoring in surgery room. Our data showed that, not only both P3 and P5 peak times themselves were increased, P3-to-P5 latency was increased as well (*Q* = 17.81, *p* = 0.003 < 0.01) (Figure 5), which suggested that the increase in P5 peak time did not entirely derive from the increase in P3 peak time. However, unlike auditory P3-to-P5 latency, visual P4-to-N6 latency was not affected by isoflurane.

## 4 Discussion

Evoked potentials are averaged EEG signal time-locked to stimulus onsets. The main purpose of our study was to examine the effect of isoflurane on AEPs/VEPs rather than the identification of their neural generators (Fullerton et al., 1987). Although we recognize that an evidenced mapping of response components with specific anatomic regions will be of great importance for understanding the neurophysiology of both sensory processing and anesthesia and allow for better comparison with human AEPs/VEPs data, it would require more fundamental studies to ensure such a mapping is solid.

### 4.1 Comparison between auditory and visual system

As far as we know, there is very little discussion on how the effect of anesthetics is different across different sensory modalities. The auditory and the visual systems have been compared from various perspectives, including cortical columnar distribution and thalamocortical transformation (Linden and Schreiner, 2003), parallel what- and where-pathways (Rauschecker, 2015), and auditory and visual scene analysis (Kondo et al., 2017). Along ascending auditory pathway, acoustic input initially coded by the cochlea is processed in multiple stations, including the brainstem, midbrain, thalamic, and cortical nucleus/regions (Malmierca and Hackett, 2012).

In comparison, visual inputs are initially coded and processed at retina, then carried to the thalamus by retinal ganglion neurons, and eventually relayed to visual cortex (Wurtz and Kandel, 2000). The findings of this study provided unprecedented evidence of how the auditory system differs from the visual system from the perspective of anesthesia tolerance. There has been a discussion on whether anesthetics cast a general suppression across different brain regions, also known as the wet blanket theory (Hudetz, 2012), or a suppression that targets specific brain regions (Sukhotinsky et al., 2007). The latter is supported by neuroimaging data, where some resting state functional MRI networks (the default mode network and the executive function network) were suppressed in propofol anesthesia, while some other networks (the auditory and the visual network) were not (Boveroux et al., 2010). However, it is also possible that higher order brain regions demonstrated larger anesthetic effects due to an accumulation along the ascending input pathway. When contrasting the auditory and visual system, one would expect to see more drastic changes in long-latency components of AEPs than VEPs of long-latency components, according to a global rather than local suppression of anesthetics, because the auditory ascending pathway requires more synaptic relays. This was not the case in our results. In fact, we observed more resistance to isoflurane in cortical AEPs than VEP using our stimulus paradigm, and one of the explanations can be that synapses along ascending auditory and visual pathways are not equivalently affected by isoflurane. However, given the complexity of comparing two sensory modalities, the stimulus repertoire needs to be extensively expanded, with varying inter-stimulus interval, intensity levels, stimulus locations, etc., before a solid conclusion can be drawn.

The same MRI study also found that an auditory contribution to the visual network was lost during propofol anesthesia (Boveroux et al., 2010), suggesting the altered functional connectivity as a new mechanism of anesthetic effect. It is also worth noting that the authors did not identify any visual contribution to the auditory network, during either awake or propofol anesthesia stages. Therefore, lateral and feedback inputs to the auditory or visual system can be a potential target in addition to the feedforward inputs for understanding the different isoflurane effect between AEPs and VEPs.

### 4.2 Effect of isoflurane on multi-modal, spectrotemporal components of evoked potentials

Among the studies comparing the effect of anesthesia on evoked potentials of more than one sensory modalities (Sebel et al., 1984; Sebel et al., 1986), the current study was the first to allow AEPs and VEPs to be derived from the same segment of EEG signal. All the evidence, including ours, suggested that VEPs were more susceptible to anesthetics than AEPs. Additionally, evidence showing that human VEPs alone were attenuated by a variety of anesthetics including isoflurane (Chi and Field, 1986; Chi et al., 1989; Chi and Field, 1990; Tenenbein et al., 2006; Sloan et al., 2010; Chitranshi et al., 2015; Ito et al., 2015; Tanaka et al., 2020). One study has showed that isoflurane and halothane, but not enflurane, suppressed VEPs recorded from visual cortex (Ogawa et al., 1992). Unsurprisingly, studies of SL-AEPs or BAEPs alone showed no anesthetic effects on peak amplitudes (Drummond et al., 1985; Manninen et al., 1985; Thornton et al., 1992; Nakagawa et al., 2006; Sloan et al., 2010; Ruebhausen et al., 2012), with the exception of sevoflurane (Nakagawa et al., 2006).

The effects of anesthetics on ML-AEP, on the other hand, has only been studied by a handful of groups. Two studies from the same group (Schwender et al., 1994a; Schwender et al., 1994b) reported that human ML-AEPs were completely absent with some anesthetic agents, including isoflurane, but unaffected with others. Another group reported similar findings with various agents in different studies (Thornton et al., 1984; Thornton et al., 1985; Thornton et al., 1986; Heneghan et al., 1987; Thornton et al., 1989). In the present study, however, even the highest concentrations of isoflurane did not achieve the absence of ML-AEPs as observed in humans. Instead, the increase of isoflurane concentration resulted in more waveform reformation rather than attenuation for ML-AEPs. This was shown by two previous reports in humans (Dutton et al., 1999; Iselin-Chaves et al., 2000) using desflurane and propofol, and in dogs (Murrell et al., 2004; Murrell et al., 2005) using sevoflurane and acepromazine.

LL-AEPs demonstrated waveform reformation with the administration of isoflurane, as well as significant decrease in the signal strength. It has been recently shown that rats P1-to-N1 amplitudes were also suppressed by isoflurane (Brewer et al., 2021). In both species, LL-AEP components seemed moderately resilient to and were not completely abolished by isoflurane as in human (Plourde and Boylan, 1991; Plourde and Picton, 1991; Simpson et al., 2002).

Finally, the short latency components found in VEPs, which were time-locked to flash onset and referred as short-latency VEPs or SL-VEPs, were indeed decreased by isoflurane and increased after isoflurane administration terminated, suggesting that these components are driven by physiological sources rather than electrical artifact.

### 4.3 Dose-dependent effect of isoflurane on sensory processing

**Figure 6:**
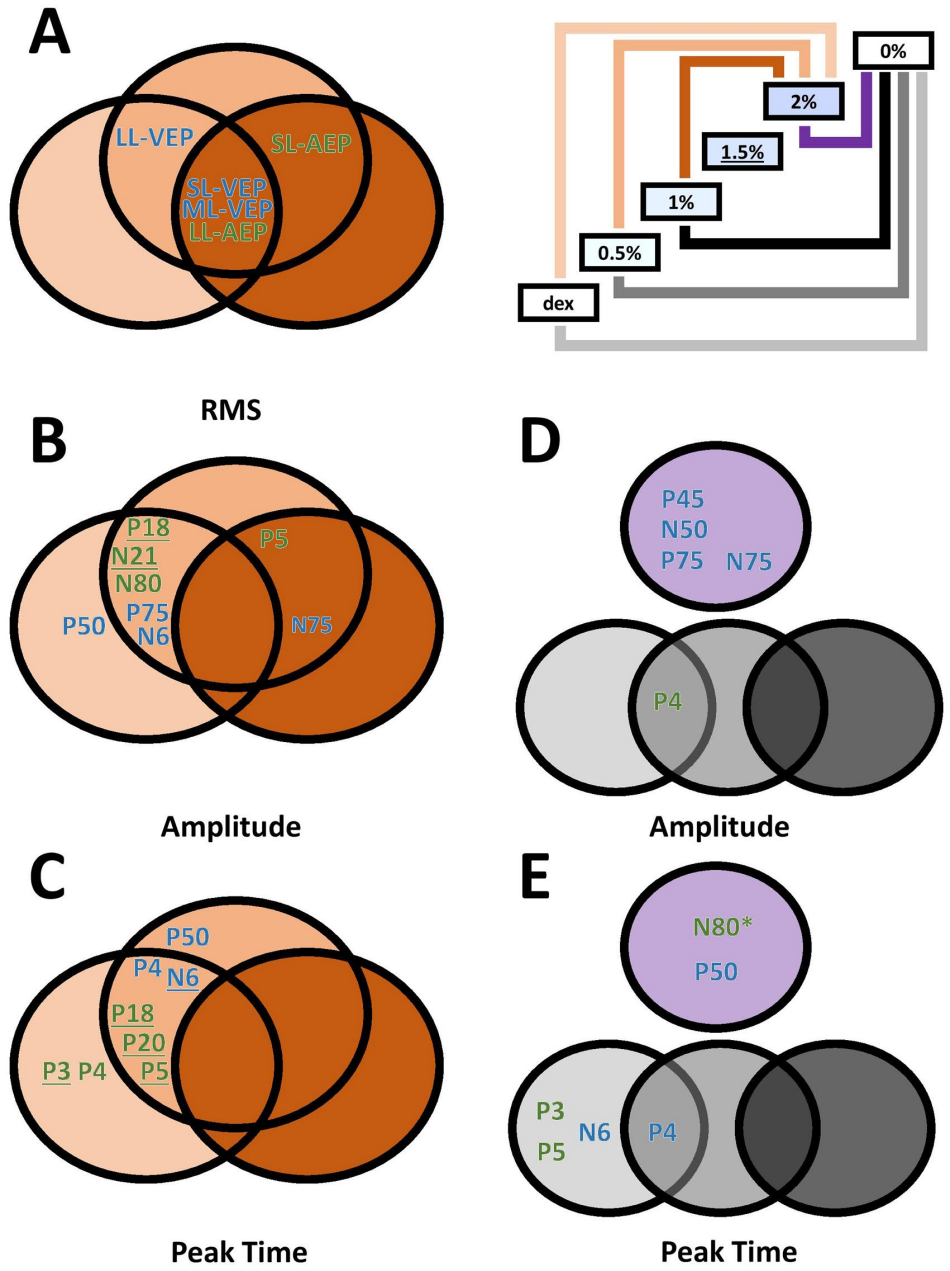
Venn diagrams of dose-dependent and wash-out effect of isoflurane in different measurements. **(A–C)** Effect of isoflurane when compared to the 2%-isoflurane blocks. Green, AEP measurements. Blue, VEP measurements. Underlined, additionally significant difference between the 1.5%-isoflurane and the dexmedetomidine blocks. **(D,E)** Wash-out effect of isoflurane during the 0%-isoflurane block. * N80 peak time also showed significant difference between the 0%- and the 1.5%-isoflurane blocks.

A secondary factor that we investigated was the relationship of isoflurane dosage and its effect on auditory and/or visually evoked potentials (Figure 6). Since the order of isoflurane concentrations was not randomized and there was no gap between two adjacent blocks for the elimination of isoflurane, we are not able to claim our findings in the context of anesthesia dosage. Rather, we used the blocks of up-stepping isoflurane concentration to manipulate the amount of the drug on an ordinal scale. It appeared that the change in most measurements did not reach the point of statistical significance until 2% isoflurane block, with several exceptions where this point was advanced to 1.5% isoflurane block (Figures 6B,C, Underscored). These observations, however, could have shed some light on the uncharted dose-dependency of isoflurane effect on AEPs and VEPs that have yet to be fully investigated.

The washout effect after the termination of isoflurane was observed. The comparison between the 2%-block and the following 0%-isoflurane block revealed several VEP measurements significantly recovered in the first 10 min after the subjects stopped receiving isoflurane, in addition to one AEP measurement (i.e., N80 peak time). The washout effect in VEP measurements may explain an earlier report of intraoperative VEP monitoring data where VEPs were not completely absent, but very unreliable (Kamio et al., 2014). On the other hand, the lack of washout effect in several AEP measurements may suggest that the effect observed during high-concentration isoflurane blocks could also be attributed to non-isoflurane factors, such as body temperature or the wear-off of dexmedetomidine.

### 4.4 Summary and implications for future studies

In this study, we examined the effect of isoflurane on auditory evoked potentials (AEPs) and visually evoked potentials (VEPs). The effect of isoflurane on signal strength prominently differentiated LL- and ML-VEPs, whereas its effect on individual peak components revealed a variety of patterns that were complicated by dose-dependency. The overall stimulus rates for both click pulses and flash pulses were higher than those commonly used, and therefore the responses were not allowed to return to baseline between stimulations. The AEPs and VEPs, as well as the isoflurane effects they revealed, were not expected to be identical with those evoked by a single impulse of stimulus, due to the non-linear interaction between the responses of two adjacent stimuli that were temporally close to each other. Our data cannot differentiate the direct effect of isoflurane on the stimulus-driven sensory pathway from its indirect effect that works through neural circuits involved in the non-linearity of the sensory system. Evidence of anesthetic effect on such a system have been previously documented, such as input–output relationship of cortical neurons (Aksenov et al., 2019) and laminar functional connectivity (Baek et al., 2022). Similarly, the periodic sensory stimuli, as used previously in assessing anesthetic effect (e.g., Sebel et al., 1986; Simpson et al., 2002), may interact with rhythmic cortical activities and therefore mediating indirect anesthetic effect. To further elucidate how different cortical modulation systems are involved in the anesthetic effects on the sensory evoked potentials, we propose the use of our stimulus paradigms with combinations of different stimulus rates (Eggermont, 1991). It will be also beneficial to incorporate other stimulation devices (e.g., LEDs-attached goggles) (Soffin et al., 2018) and recording media (e.g., cup electrodes) (Santangelo et al., 2018).

The use of sensory evoked potentials in intraoperative monitoring is promising but requires improvements in protocols and devices. Since it allows for a continuous examination on the functional integrity along the ascending sensory pathways, it may serve as an augmentation to current EEG-based anesthesia monitoring such as bispectral index (National Institute for Clinical Excellence, 2012).

## Data Availability

The raw data supporting the conclusions of this article will be made available by the authors, without undue reservation.
