# Supplementary material for: Differential effects of isoflurane on auditory and visually evoked potentials in the cat
Source: Front Syst Neurosci. 2024 Dec 4;18:1367525. doi: 10.3389/fnsys.2024.1367525 (PMC11656312; doi:10.3389/fnsys.2024.1367525)
Supplement: Supplementary file 1 [file Data_Sheet_1.pdf]

Supplementary Materials

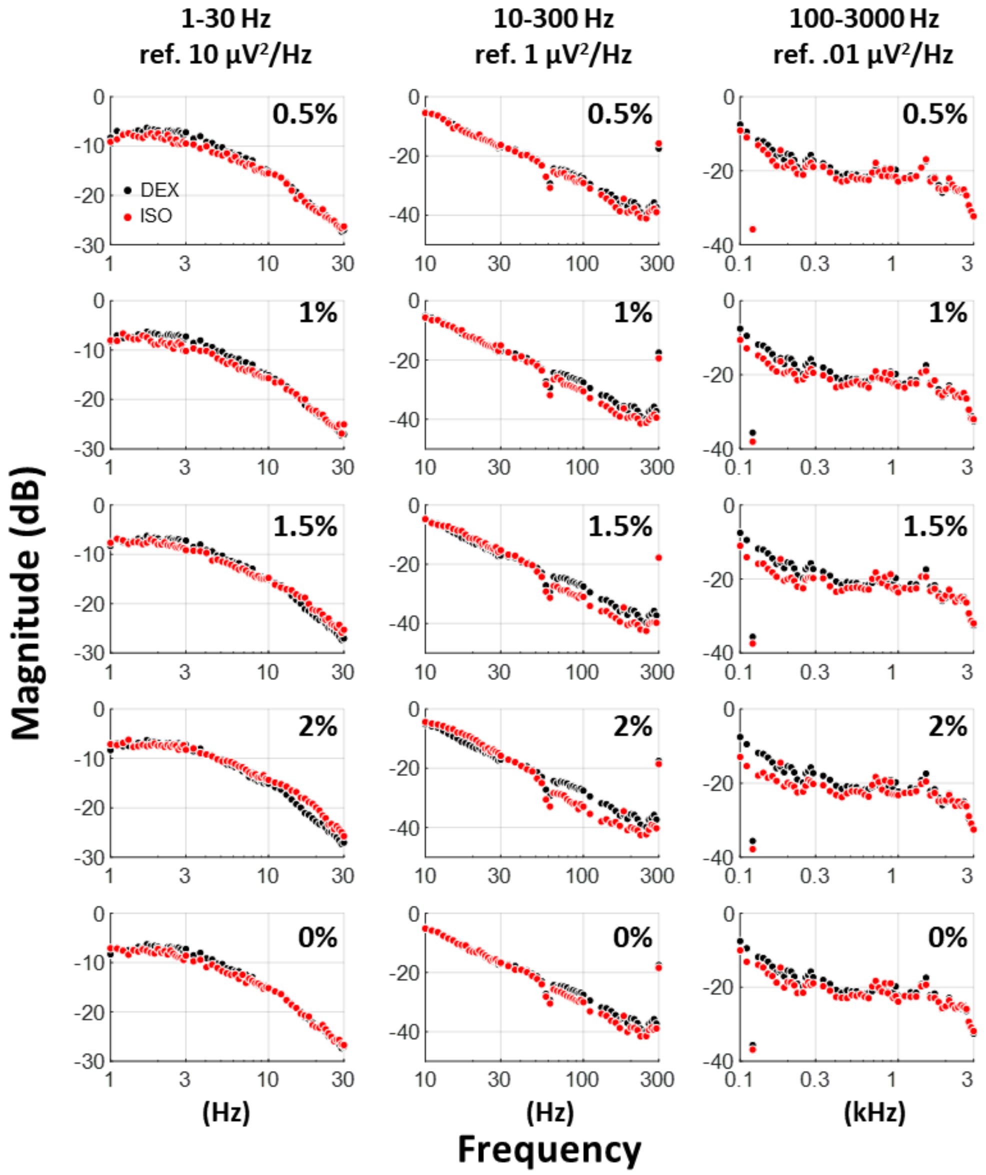

Supplementary Figure 1. Welch's Spectrum of EEG signal under different isoflurane conditions. Welch's spectrum was estimated at frequencies at logarithmic scale between 1 Hz and 3000 Hz. Medians across 10 repeats and 5 subjects for each block was plot as the function of frequency. For the display purpose, the power density values were normalized separately for three frequency bands to 10 uV^2/Hz (1-30 Hz, left), 1 uV^2/Hz (10-300 Hz, middle), and 0.01 uV^2/Hz (10-300 Hz, right). The concentration of isoflurane was indicated on the top-right corner of each panel and the Dex-only block plotted as a control. The power spectrum of the Dex block was plotted in black for all different. The middle and right columns demonstrated a prominent suppressive effect of isoflurane on EEG power density between 50 Hz and 300 Hz, especially for the 2%-isoflurane block. A less prominent suppression around 3 Hz (theta band) was observed for low isoflurane concentrations while an enhancement around 20 Hz (beta band) was observed for the 2%-isoflurane block.

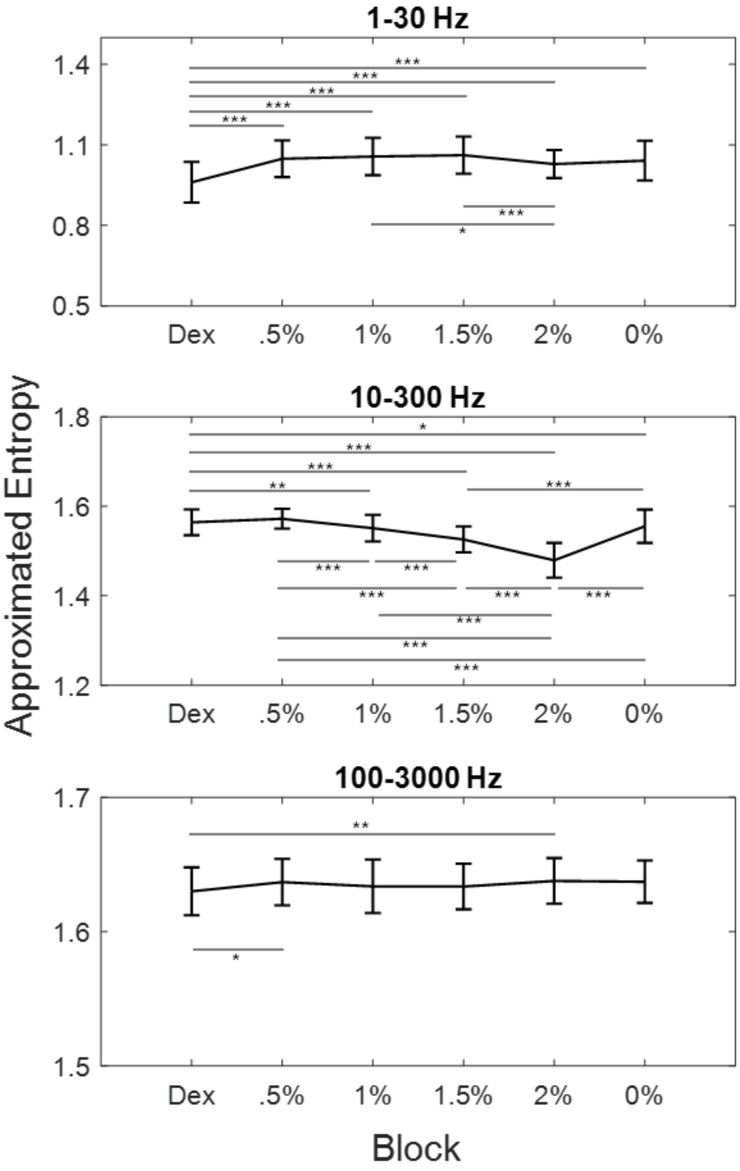

Supplementary Figure 2. Approximate entropy in time domain for bandpass filtered EEG signals. Each one-minute EEG signal was down-sampled to about 128 Hz and divided into 7 segments of 1000 data points. Approximate entropy was estimated for each segment, about 7.8 second, and in total 350 approximate entropy values were obtained for each block. Medians of all 6 isoflurane conditions were compared using Mann-Whittney U tests with Beefaroni's correction. Error bar, inter-quartile range. *, $p < 0.05$. **, $p < 0.01$. ***, $p < 0.001$.

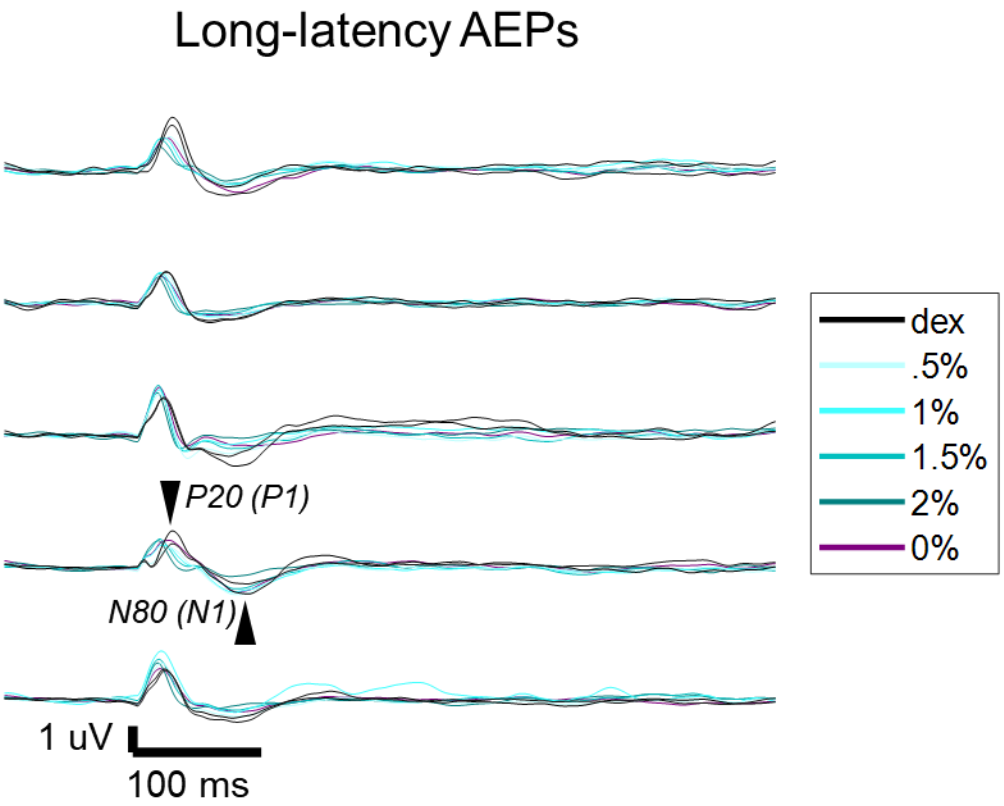

Supplementary Figure 3. The waveforms of long-latency AEPs.
Each of the five rows demonstrates the waveforms for Subject 1 to Subject 5, from bottom to top. Noted that different time and amplitude scales were used. The isoflurane/anesthesia treatment is color-coded. Black, baseline block with only dexmedetomidine (i.e., Dex) administrated. Blue with light-to-dark shading, the isoflurane blocks with up-stepping concentrations. Purple, the block after isoflurane stops. Arrows and labels indicate the selected peak components for further analysis.

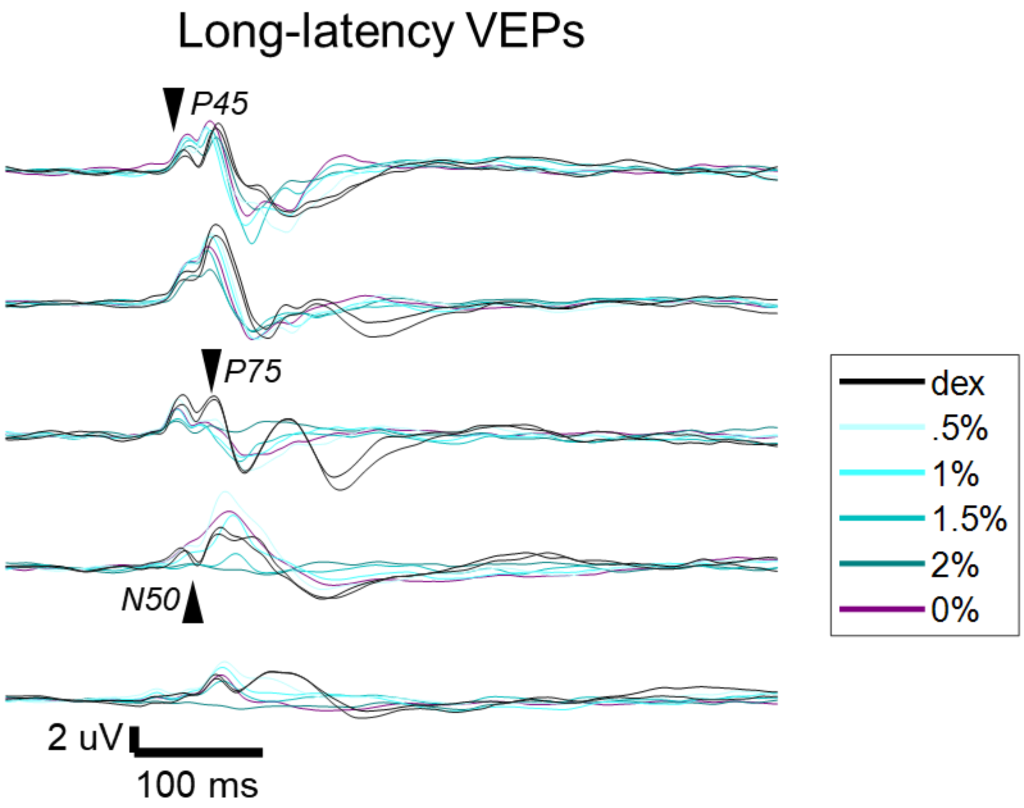

Supplementary Figure 4. The waveforms of long-latency VEPs.
For conventions, see **Supplementary Fig. 3**.

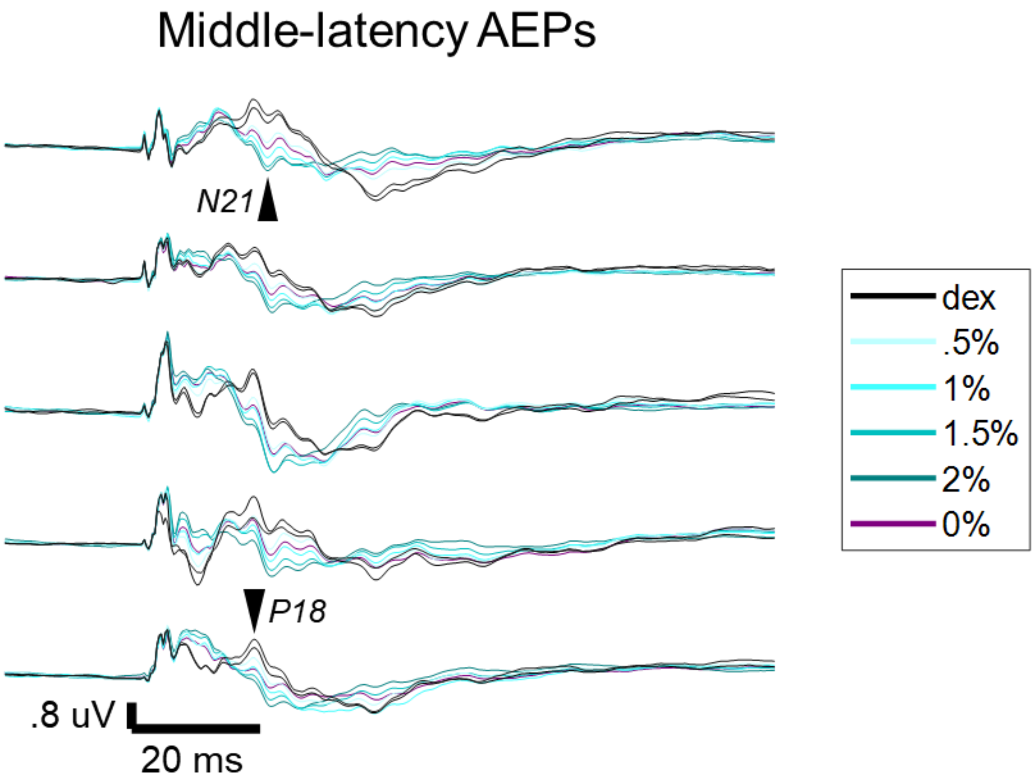

Supplementary Figure 5. The waveforms of middle-latency AEPs.
For conventions, see **Supplementary Fig. 3**.

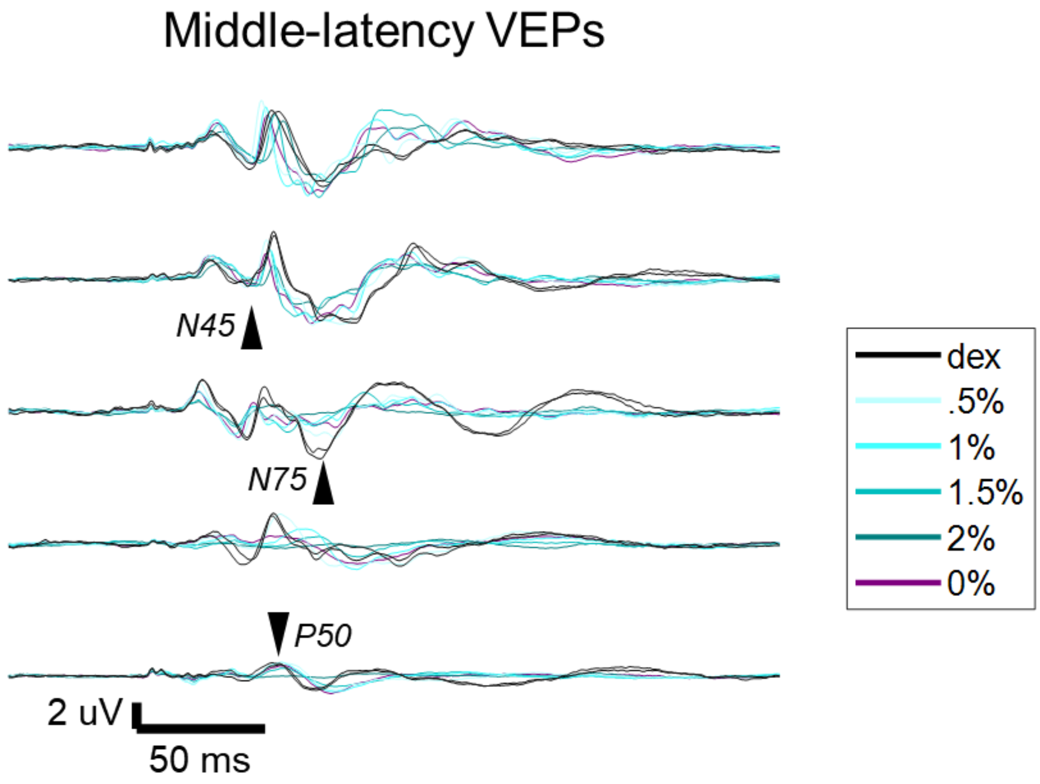

Supplementary Figure 6. The waveforms of middle-latency VEPs.
For conventions, see **Supplementary Fig. 3**.

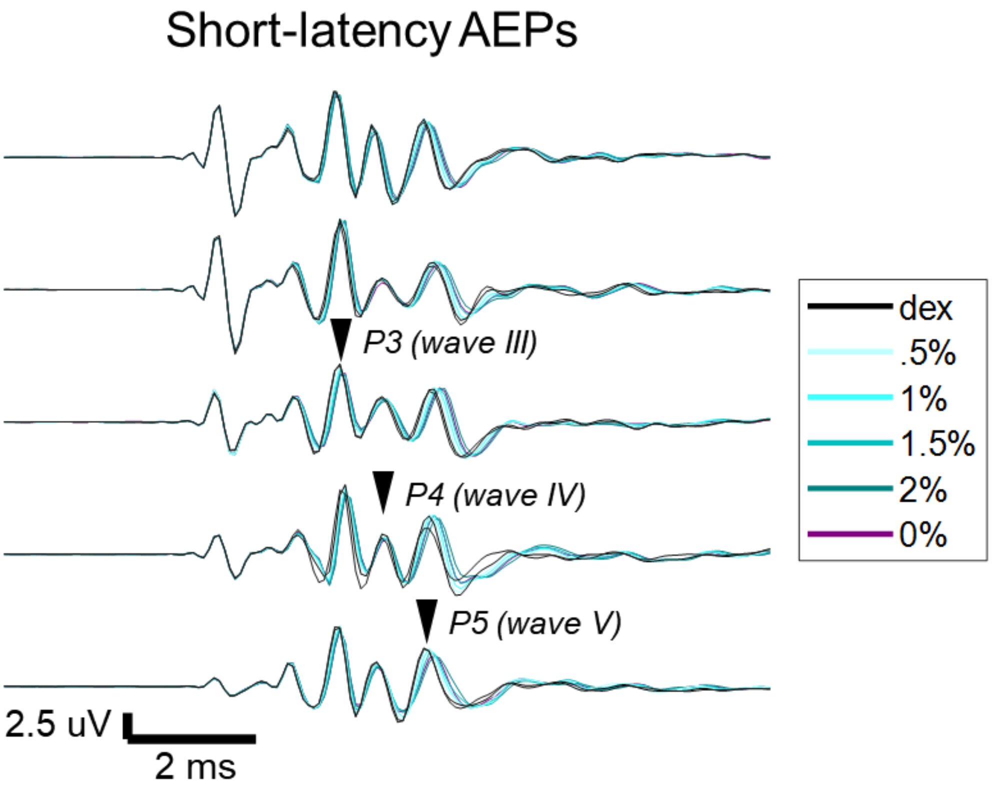

Supplementary Figure 7. The waveforms of short-latency AEPs.
For conventions, see **Supplementary Fig. 3**.

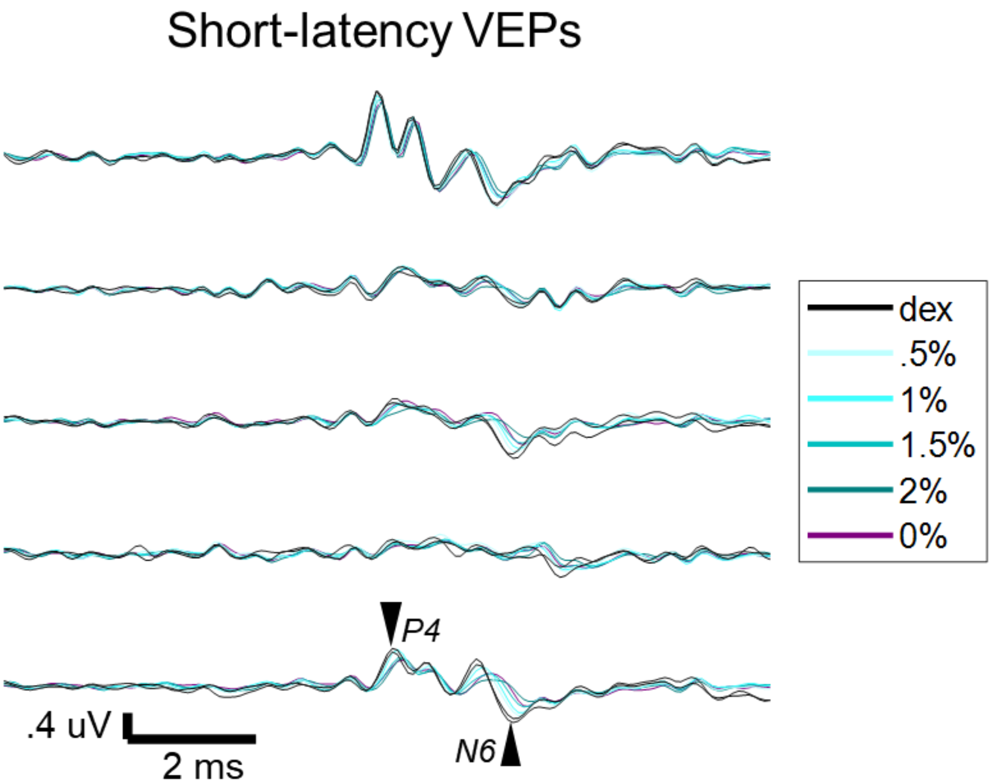

Supplementary Figure 8. The waveforms of short-latency VEPs.
For conventions, see **Supplementary Fig. 3**.

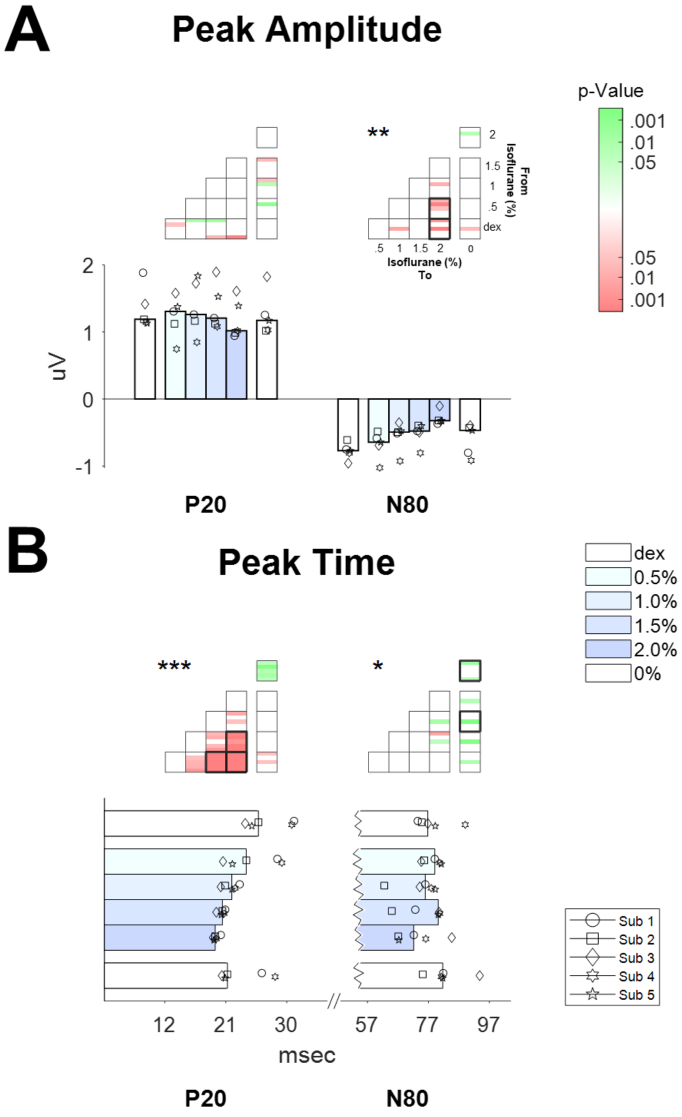

Supplementary Figure 9. Effect of isoflurane on the long-latency (LL-) AEP components P20 and N80. For conventions, see **Fig. 3** legend. Dots with different shapes, data of individual subject. The heat maps on the top show the P-values from multiple comparison tests for individual subject, between the conditions labeled on the right and at the bottom. Green, increase. Red, decrease. Black border, a significant pair in the multiple comparison tests from the group statistics ($p < .05$). We only quantified the first two peak components (i.e., the P20 and the N80, also known as P1 and N1) from LL-AEPs, as the components later were not consistently accessible. **A) Left and B) Left,** only P20 peak time but not peak amplitude was significantly decreased by isoflurane ($Q = 24.54$, $p < .001$). Multiple comparison test showed a significant decrease in P20 peak time in the 2%-isoflurane block, when compared to the Dex block ($p < .001$) or the 0.5%-isoflurane blocks ($p < .01$), but not the 1%-isoflurane block. This pattern was inversed from what we observed with the RMS values, in which case the decrease was only significant from the 2%- to the 1%-isoflurane blocks. Also, the decrease from the Dex to the 1.5%-isoflurane block was also significant ($p < .01$), suggesting that P20 peak time could be affected by isoflurane of a lower concentration than the overall RMS value. **A) Right and B) Right,** we also examined the N80 component, and we found its amplitude was significantly suppressed by isoflurane ($Q = 18.14$, $p < .01$). Same as in P20 peak time, N80 amplitude was significantly lower in the 2%-isoflurane block when compared to the Dex ($p < .01$) or the 0.5%-isoflurane block ($p < .01$), suggesting that P20 peak time and N80 amplitude may have an innate conjugation, but not with the overall RMS value. There was also a block effect on N80 peak time with a weak statistical significance ($Q = 12.86$, $p < .05$). However, multiple comparison test only revealed significant difference ($p < .01$) between the 0%- and the 2%-, and between the 0%- and the 1%-isoflurane block. Our findings from the P20 and the N80 components suggested that the small suppression in the signal strength of LL-AEPs may originate from the decrease in both N80 amplitudes and P20 peak time.

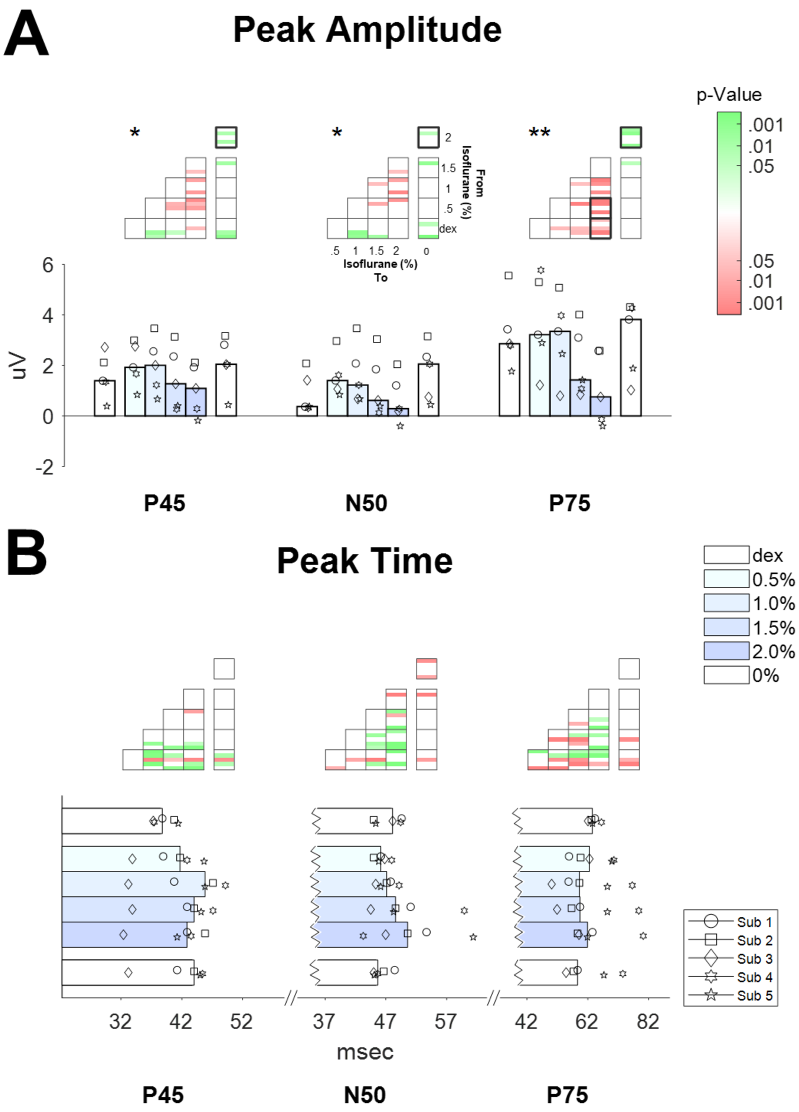

Supplementary Figure 10. Effect of isoflurane on the long-latency (LL-) VEP components P45, N50, and P75. For conventions, see **Fig. 3 and Supplementary Figure 2** legend. **A)** The amplitude of the P75 component from LL-VEPs was significantly attenuated by isoflurane in general (**right**), with a slight increase during the 0.5%-isoflurane block. We found the block effect of isoflurane on P75 amplitude with medium statistical significance ($Q = 17.57$, $p < .01$). Multiple comparison test showed that a significant decrease occurred during the 2%-isoflurane block, when compared to the Dex ($p < .05$) or the 0.5%-isoflurane blocks ($p < .01$). A similar pattern can be found in the P45 (**left**) and the N50 components (**middle**), despite of the identification of these two were particularly difficult in subject No. 5 with a low signal-to-noise-ratio in its atypical waveform. Except for a slight increase during the 0.5%-isoflurane block, both P45 amplitude ($Q = 12.09$, $p < .05$) and N50 amplitude ($Q = 14.71$, $p < .05$) were overall attenuated by isoflurane. It is worth noting that multiple comparison tests revealed significant washout effect ($p < .05$) in the 0%-isoflurane block in comparison with the 2%-isoflurane block for all three peak amplitudes from LL-VEPs. **B)** Meanwhile, the peak time of the three showed no block effect of significance. These findings suggested that the suppression of RMS values from LL-VEPs was contributed by all three components we selected.

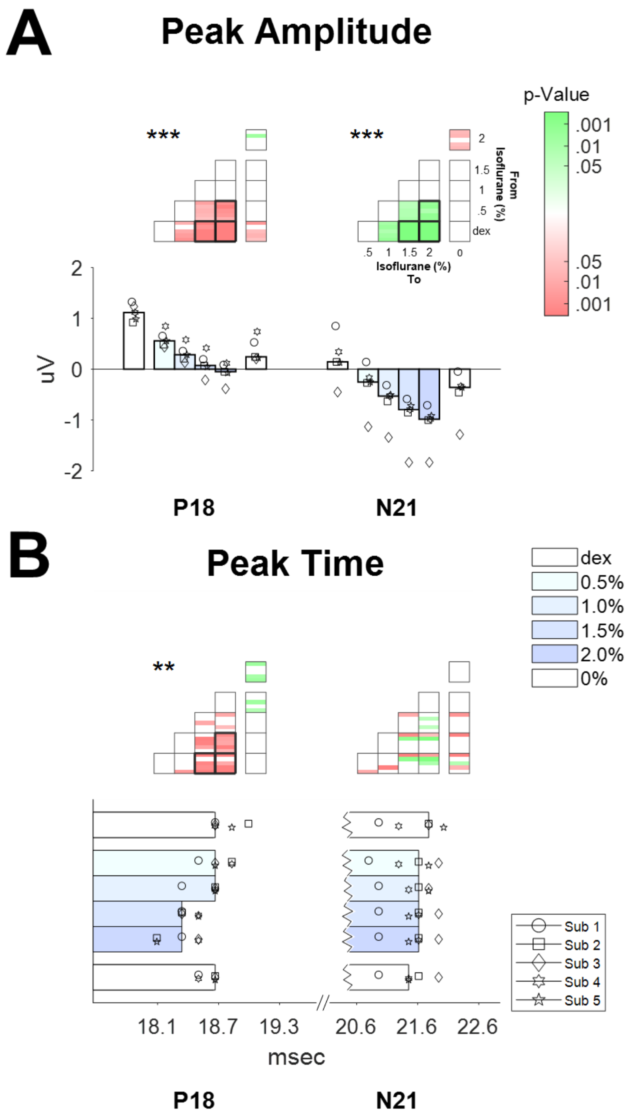

Supplementary Figure 11. Effect of isoflurane on the middle-latency (ML-) AEP components P18 and P21. For conventions, see **Fig. 3 and Supplementary Figure 2** legend. Whereas the RMS values of ML-AEPs were not suppressed by isoflurane, the waveforms of ML-AEPs were drastically altered. A number of peak components can be derived from ML-AEPs, but we only selected the amplitudes and peak times of the P18 and the N21 component for further quantitative analysis, as these two appeared to have the most representative change in waveform. **A)** Both peak amplitudes significantly shifted towards more negative values, that is, in the case of P18 amplitude (**left**), towards zero ($Q = 24.54$, $p < .001$), and, in the case of N21 amplitude (**right**), away from zero ($Q = 25.00$, $p < .001$). Multiple comparison tests showed that for both P18 and N21 amplitudes the differences were significant, between the 2%-isoflurane and the Dex blocks ($p < .001$), between the 2%- and the 0.5%-isoflurane blocks ($p < .01$), and between the 1.5%-isoflurane and the Dex blocks ($p < .01$). **B)** Additionally, P18 peak time (**left**) demonstrated statistically significant decrease ($Q = 19.80$, $p < .01$). Multiple comparison test showed significant decrease between the 2%-isoflurane and the Dex blocks ($p < .01$), between the 2%- and the 0.5%-isoflurane blocks ($p < .05$), and between the 1.5%-isoflurane and the Dex blocks ($p < .01$). However, N21 peak time was unaffected (**right**). It is worth pointing out that the same positive findings observed as in the group statistics were followed by each of five subjects, with the only exception being subject No. 5 only for P18 peak time. Furthermore, the amount of peak amplitude shift appeared to be in linear relationship with the concentration of isoflurane, which was not often observed in the other components or the RMS values.

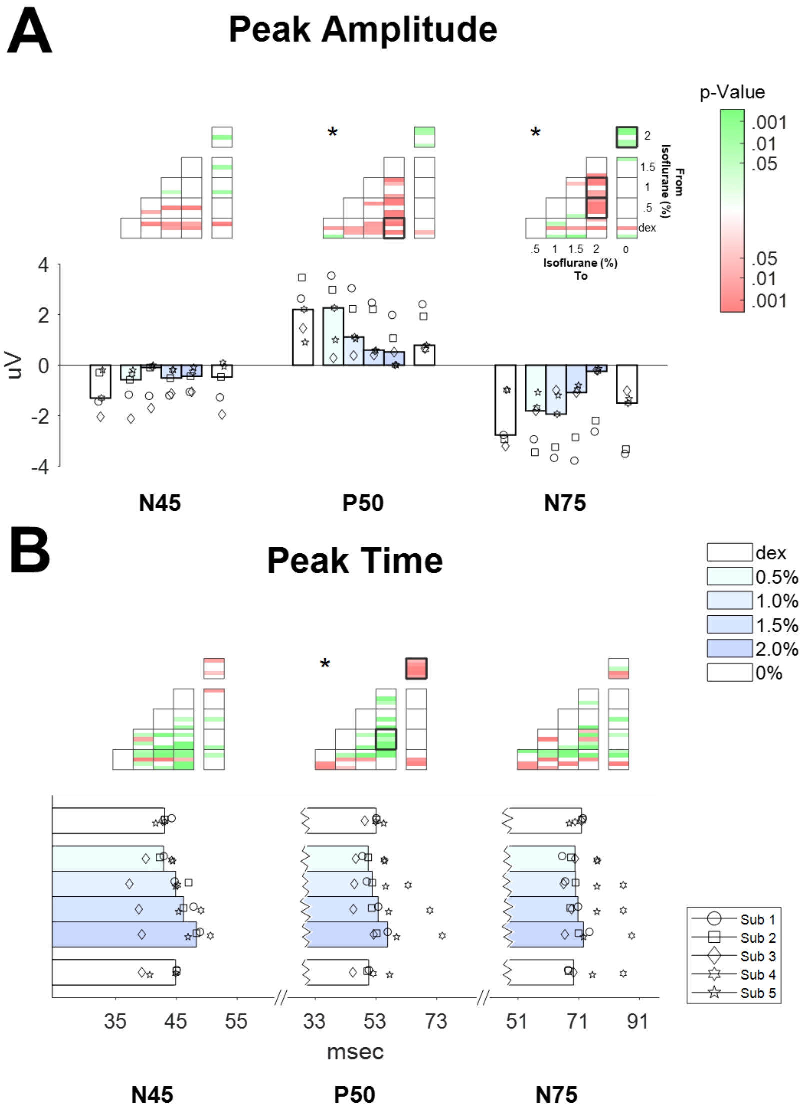

Supplementary Figure 12. Effect of isoflurane on the middle-latency (ML-) VEP components N45, P50, and N75. For conventions, see **Fig. 3 and Supplementary Figure 2** legend. We quantified three peak components from ML-VEPs with peak times corresponding to those from LL-VEPs (see **Supplementary Fig. 2**) but inverted polarities. **A)** N75 amplitude (**right**), like the P75 amplitude from LL-VEPs, was also significantly decreased (Q = 12.54, p < .05), but the following multiple comparison test showed statistical significance in the 2%-isoflurane block against the 0.5%- or the 1%-isoflurane block (p < .05), but not the Dex block. P50 amplitude (**middle**) was also significantly decreased (Q = 12.31, p < .05), but instead of the recovery effect found in N50 amplitude from LL-VEPs, the following multiple comparison test revealed a significance decrease in P50 amplitude from the Dex to the 2%-isoflurane block (p < .05). Unlike the P45 amplitude from LL-VEPs, the effect of isoflurane on N45 amplitude (**left**) was not statistically significant. **B)** Although none of the three peak times from LL-VEPs were affected by isoflurane, P50 peak time from ML-VEPs (**middle**) was significantly increased (Q = 12.70, p < .05). Multiple comparison test showed a significant increase from the 0.5%- to the 2%-isoflurane blocks (p < .05) and a significant decrease from the 2%- to the 0%-isoflurane blocks (p < .05). Together with what we found from LL-VEPs, these results suggested that the effect of isoflurane on VEPs could majorly reside in the spectral range that is overlapped between LL-VEPs and ML-VEPs, but the contribution from the non-overlapping spectral range may have also presented.

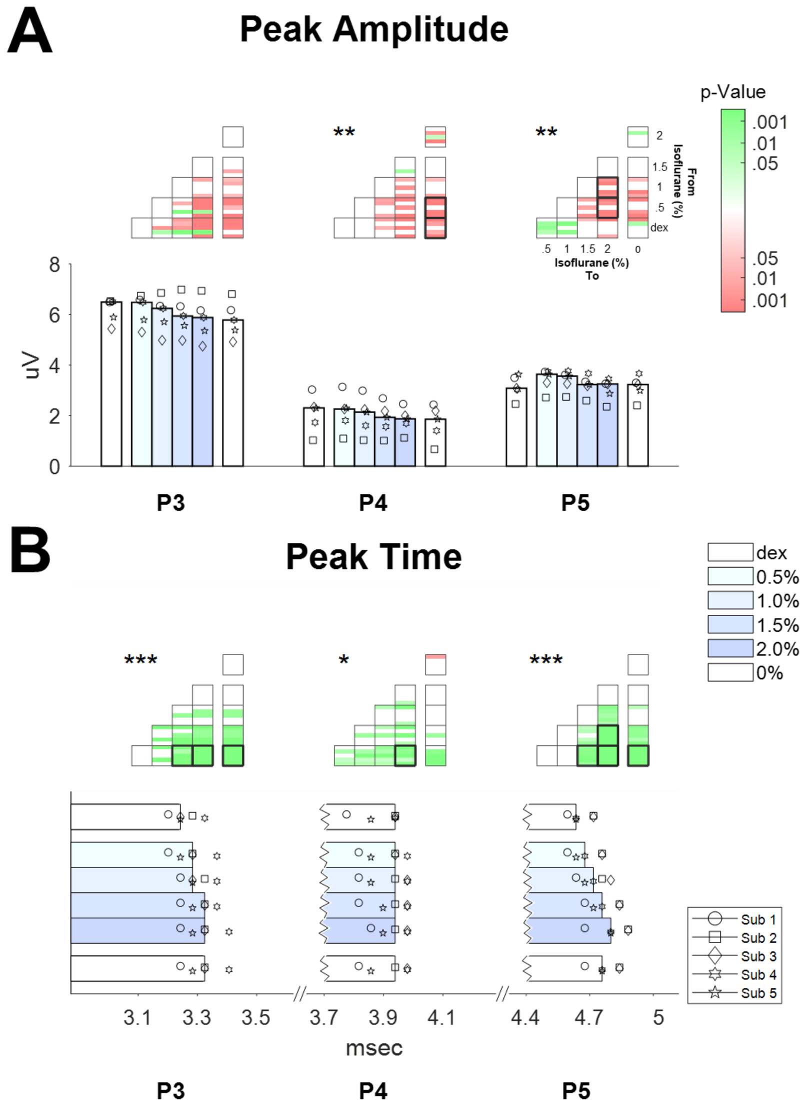

Supplementary Figure 13. Effect of isoflurane on the short-latency (SL-) AEP components P3, P4, and P5. For conventions, see **Fig. 3 and Supplementary Figure 2** legend. Among all five waves in SL-AEPs or BAEPs, wave III (P3) and V (P5) were most frequently reported in the literature. Here, we quantified P3 (wave III), P4 (wave IV), and P5 (wave V) amplitude and peak time for further analysis. **A)** Isoflurane significantly reduced P4 amplitude ($Q = 16.89$, $p < .01$, **middle**) and P5 amplitudes ($Q = 17.11$, $p < .01$, **right**) but not P3 amplitudes (**left**). Multiple comparison tests showed that only P5 amplitude was significantly lower in the 2%-isoflurane block when compared to the 0.5%- or the 1%-isoflurane block ($p < .05$). Decrease in P4 amplitude was observed in the 2%-isoflurane block but did not reach statistical significance. The effect of isoflurane on P4 amplitude was persistent after isoflurane was stopped during the 0%-isoflurane block when compared to the Dex ($p < .05$) or the 0.5%-isoflurane block ($p < .01$). **B)** As for peak times, all of the three components were significantly affected, which includes P3 peak time ($Q = 21.21$, $p < .01$, **left**), P4 peak time ($Q = 13.52$, $p < .05$, **middle**), and P5 peak time ($Q = 24.15$, $p < .001$, **right**). They all demonstrated a significant increase from the Dex to the 2%-isoflurane block, which includes P3 peak time ($p < .01$, **left**), P4 peak time ($p < .05$, **middle**), and P5 peak time ($p < .001$, **right**), as revealed by multiple comparison tests. For the P3 and the P5 components, the increase in peak times from the Dex baseline level reached statistical significance in the 1.5%-isoflurane block ($p < .05$), which suggested that these two are likely more sensitive to isoflurane than P4 peak time. The increases were also persistent after isoflurane termination during the 0%-isoflurane block for both P3 peak time ($p < .01$) and P5 peak time ($p < .05$). P5 peak time also showed a significant difference between the 0.5%- and the 2%-isoflurane blocks ($p < .01$). In short, we found the P5 component most sensitive to isoflurane in SL-AEPs, in terms of both peak amplitude and peak time. The other components, such as the P3 and the P4 components, were affected by isoflurane as well. However, we don't consider the effect size observed in the current study, especially for peak amplitudes, was substantially larger than those reported previously. Rather, the statistically positive change may be due to low signal noise and hence stronger statistical power.

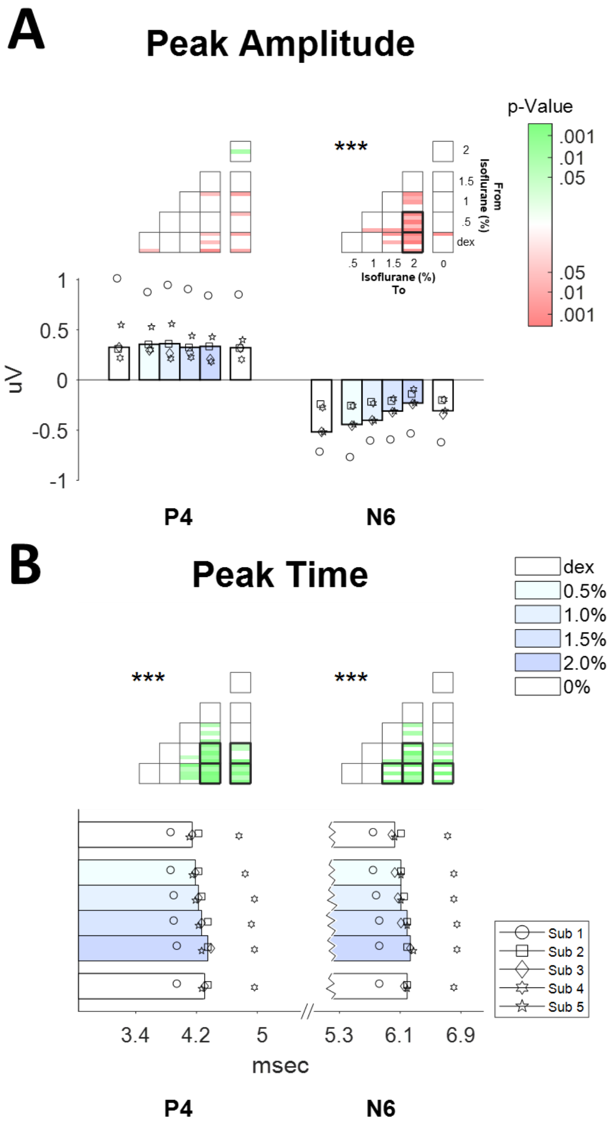

Supplementary Figure 14. Effect of isoflurane on short latency (SL-) VEP components P4 and N6. For conventions, see **Fig. 3 and Supplementary Figure 2** legend. We only quantified the P4 and the N6 components from SL-VEPs, as the other peak components cannot be consistently seen in all subjects. **A)** While P4 amplitude seemed to be spared by isoflurane, N6 amplitude was systematically attenuated by isoflurane (Q = 22.94, p < .001). Multiple comparison test revealed that the N6 amplitude in the 2%-isoflurane block was significantly lower than the Dex or the 0.5%-isoflurane blocks (p < .01). **B)** Isoflurane also significantly increased both P4 (Q = 22.90, p < .001) and N6 (Q = 22.63, p < .001) peak times. Compared to the Dex block, P4 peak time was significantly increased in the 2%-isoflurane block (p < .01) and the effect was persistent after isoflurane termination during the 0%-isoflurane block (p < .05), whereas N6 peak time was significantly increased in not only the 2%- (p < .01) but also the 1.5%-isoflurane block (p < .05) with the effect persistent during the 0%-isoflurane block (p < .05) as well. Multiple comparison tests also revealed significant difference between the 2%- and the 0.5%-isoflurane blocks as well (p < .05). However, unlike auditory P3-to-P5 latency, visual P4-to-N6 latency was not affected by isoflurane.
